# Testing the reported long-term advantages of protein-fortified human milk in very low birth weight neonates

**DOI:** 10.3389/fped.2024.1406637

**Published:** 2024-05-24

**Authors:** Augusto Biasini, Erica Neri, Marcello Stella, Laura Malaigia, Elisa Mariani, Vittoria Rizzo, Francesca Agostini

**Affiliations:** ^1^ Donor Human Milk Bank Italian Association (AIBLUD), Milan, Italy; ^2^ Department of Psychology “Renzo Canestrari”, University of Bologna, Bologna, Italy; ^3^ Pediatric and Neonatal Intensive Care Unit, Maurizio Bufalini Hospital, Cesena, Italy

**Keywords:** very low birth weight infant, protein intake, human milk, long term neurological advantages, psychological outcomes

## Abstract

Preterm infants are at-risk for extrauterine growth restriction and downward percentile-crossing between birth and discharge. Increased energy and protein intake through fortification of human milk during the first weeks of life has been associated with improved short-term growth and better developmental outcomes. The aim of this study was to evaluate whether these benefits persist up to children school age. The study was designed as an observational study. During hospitalization, 22 very low birth weight preterm infants were fed with increasing protein fortification of human milk (protein supplemented group, PSG). As a control group (CG), 11 preterm infants were fed with standard nutrition regimen. At children school age (9–11 years), we assessed anthropometric data (weight, height, BMI), global health (renal function), and specific psychological outcomes (Child Behavior Checklist 6–18). A global homogeneity between CG and PSG groups emerged: we found no significant differences in weight, height, and BMI, nor in internalizing symptom outcomes (all *p*s > 0.05). However, mothers reported significantly higher externalizing symptoms for the PSG infants compared to CG infants. Therefore, neonatal enteral protein supplementation in very low birth weight preterm infants leads to no positive nor adverse consequences in long-term assessment, suggesting that benefits are restricted to the neonatal term and first years of age.

## 1 Introduction

Preterm infants frequently develop postnatal growth restriction during their hospitalization in Neonatal Intensive Care Units (1, 2). This could lead to negative consequences, like a higher risk of developing metabolic disorders such as obesity, diabetes and cardiovascular disease and increased motor, cognitive and socio-emotional impairment from school age to adulthood (3–5).

The European Society of Paediatric Gastroenterology, Hepatology and Nutrition (ESPGHAN) has recommended a high protein intake in preterm babies born between 26 and 30 weeks of gestational age (3.8–4.4 g/kg/d, energy ratio 3.4) given its positively association with lean body mass growth (6). Increased energy and protein intake through fortification of human milk during the first weeks of life have been associated with improved short-term growth from infancy to adolescence in preterm and ELBW babies (7, 8). Furthermore, high-caloric nutrition has been associated with increased total brain and basal nuclei volumes as revealed by MR metric (9). Previous studies also described better developmental outcomes during the first 24 months of infant's corrected age, with a higher improvement especially for severe preterm infants, such as ELBW and SGA ones (8, 10–12).

Despite the rapid body mass index (BMI) gain and linear growth may improve cognitive development, they are accompanied by an increased risk of developing metabolic and cardiovascular disease in adulthood (13).

To our knowledge, up to now the literature mainly has focused on early years post-discharge, while the investigation of long-term impact of protein-fortification in preterm children is lacking. Therefore, the aim of this study was to further investigate the potential benefits of feeding preterm infants with human fortified milk; specifically, we hypothesized that this treatment would have been significantly associated to long term advantages in preterm auxological, and psychological outcomes evaluated at child's school age.

## 2 Method

This study is part of a wider research aimed to assess the effect of milk fortification on preterm infant outcomes in first two years of corrected age (8, 10–12). So, the present study represents a follow up with the objective to explore the state of children school age.

According to methodology explained on our previous published studies (8, 10–12), we recruited 61 preterm neonates born between January 2010 and March 2011 admitted at the level III Neonatal Intensive Care Unit of Bufalini Hospital, Cesena (Italy).

Inclusion criteria for the recruitment were: birth weight (BW) < 1,500 g (Very Low Birth Weight-VLBW), gestational age (GA) < 32 weeks, exclusive human milk feeding (own mother's milk or donor milk from the local human milk bank) during NICU stay, absence of sepsis, intraventricular hemorrhage grade 3 or 4, periventricular leukomalacia, retinopathy of prematurity grade 3 plus disease, necrotizing enterocolitis (NEC) Bell's stage 2 during hospitalization.

All newborns were fed with fresh maternal milk (OMM) and/or donor human milk (DHM), supplemented according to different protein fortification regimens, starting at post-natal day 1.

During hospitalization, neonates were randomly allocated to groups, according to a daily scheduled assignment: infants born at odd and even days were allocated in Protein Supplemented Group (PSG) and Control Group (CG), respectively.

PSG group included 34 neonates (19 ELBW, 15 VLBW), that were fed with fresh OMM or DM since their first day of life. Feeds started at volumes of 10–15 ml/kg/day, divided in 10 meals; when an adequate feeding tolerance was established, feeds were increased by 20–25 ml/kg/die.

Twenty-seven infants (13 ELBW, 14 VLBW) were included in the Control Group (CG): according to Standard Nutrition Protocol, administered by combined enteral and parenteral nutrition according to the European Society of Pediatric Gastroenterology and Nutrition (ESPGHAN) guidelines, they received 4.8 g of protein/kg/day and 3.5 g of protein/kg/day, respectively.

It was used “adjustment fortification” with level of fortification based on blood urea level. Assuming protein human milk content of 0.8–1.1 gr/dl and the diet volume intake of 160 ml/kg^−1^ per day, the max protein intake should have been about 3,5 g kg^−1^ per day in the control group whereas the protein supplemented group (PSG) would achieve a protein intake of 4.8 g/kg^−1^ per day receiving supplemented protein intake by graded amounts of protein. The end of the study was set at the time of discharge, transfer or when the baby was able to ingest >50% of his prescribed quantity directly from the breast of his/her mother.

In both groups, about 60% of milk was provided by the infants’ own mother (OMM) and 40% was pasteurized donor milk from the hospital's milk bank (PDM). At discharge, 62.5% of all preterm infants included in the study were fed exclusively with OMM. More specific information on the kind of fortification and effects on brief term infant development were described in our previous works (11, 12).

The results of assessment during first two years were already published (8, 11, 12). The present study focused on a first assessment implemented when children were 6 years of age. Renal function of all children was evaluated by hospital pediatricians. The assessment regarded primarily PSG children in order to verify if the augmented protein in the neonatal period was adequate and if the possible transitional increase of glomerular filtration rate would have been a normal adaptative mechanism without any consequences for future kidney activity.

Subsequently, all children were assessed at about 10 years of age (mean 9.80 ± 0.59; range 8.9–10.8 years). We chose this age range to ensure that children data would not be influenced by adjustment to transition from pre-school to primary school, and also to prevent possible bias related to physiological changes occurring with the onset of puberty period.

All families were contacted through a phone call, to propose this follow-up research in February 2020, scheduling planned assessment in spring 2020. In order to facilitate families’ participation, given the restriction measures related to Covid-19 pandemic, the assessment was organized sending the families by mail a booklet including a written informed consent, a form regarding on parental sociodemographic characteristics, anthropometric data (weight and height) and a questionnaire about parental perception of the child behavior. Parents were asked to fulfill and send back by mail in 15 days.

Among the 61 recruited families for previous studies (11, 12), only 33 agreed to maintain their participation to the study. Reasons of drop out were inability to accommodate into time schedule of the study or no interest into the study. The final samples included 22 (12 ELBW, 10 VLBW) and 11 (4 ELBW, 7 VLBW) families of children of PSG and CG groups (64.7% and 40% of the initial study group, respectively).

The study was conducted according to the guidelines of the Declaration of Helsinki and ethically reviewed and approved by the Head of the NICU at the beginning of original study in 2009.

Anthropometric data on children height and weight given by parents were later correlated to Neonatal Anthropometric INeS Charts to detect percentiles and Z-scores for corrected age in the neonatal period (14). Data related to study group subjects, ages 2–10 years, were analyzed through the PediTools software, a clinical tool for Pediatricians based on CDC growth charts that enables determination of growth metric percentiles and Z-scores of children and adolescents from 2 to 20 years of age. Included in the metrics are calculation of Weight-for-age, Stature-for-age, and BMI-for-age (15). We used BMI-for-age-Z-score in childhood, according to the World Health Organization (WHO), as it represents the most widely available measure of adiposity and a predictor for overweight and obesity in adulthood. According to WHO definition, we considered obesity as BMI –for-age-Z score >3.0 and overweight by a BMI-for –age Z score >2.

Regarding child psychological outcomes through parental perception of the child, both parents fulfilled Child Behavior Checklist 6–18 (CBCL), a well-established parent-reported measure of children's emotional and behavioral functioning (16, 17). The CBCL includes 113 items describing the presence and the frequency of a specific behavior. Parents were asked to indicate how accurately each item applied to their child according to a three-point Likert scale (0, not true; 1, sometimes true; 2, very true). Scores of each item are allocated into 8 syndrome areas (anxious/depressed, withdrawn, somatic complaints, social problems, thought problems, attention problems, rule-breaking behaviour, aggressive behaviour) and summed into 2 main scales: internalizing and externalizing symptom scales. Cut off scores are considered clinically significant when the T-score is 65 or above (16, 17).

Statistical analyses were performed using the Statistical Package for Social Science software (SPSS version 21.0). Significant results were considered when *p* values were lower than 0.05.

At a preliminary level, homogeneity on clinical and sociodemographic variables between drop out and included participants groups, and between PSG and CG ones, were tested by Pearson Chi2 and Student t-test analyses for categorical and continuous variables, respectively. Given the small sample size, clinical and sociodemographic variables were included in analyses only in case of significant differences in their distribution in the two groups.

The significance of differences in anthropometric data (weight, height and BMI) reported for the two study groups (PSG and CG) was tested through unpaired t-tests performed between the two groups. Furthermore, we run unpaired *t*-tests to investigate possible differences between groups on CBCL scores, separately, for mothers and fathers.

## 3 Results

No statistical differences on clinical and sociodemographic variables among drop out infants and those included in the study emerged (all *p*s > 0.05). When we assessed clinical and sociodemographic variables between PSG and CG groups, a globally homogeneity emerged (all *p*s > 0.05) (Table 1).

**Table 1:** Child and parental characteristics according to group.

	PSG (*n* = 22)	CG (*n* = 11)	t/*x*^2^	*p*
Neonatal Variables				
Birth weight, grams, mean (SD)	981.82 (181.56)	1,028.64 (158.48)	0.727	0.473
Gestational age, weeks, mean (SD)	28.08 (2.03)	29.07 (1.52)	1.435	0.161
CRIB score, mean (SD)	2.59 (2.11)	1.73 (1.27)	−1.244	0.233
SGA, *n* (%)	7 (31.8%)	3 (27.3)	0.072	0.789
Gender, *n* (%)			0.243	0.622
Male	12 (54.50)	5 (45.50)		
Female	10 (45.50)	6 (54.50)		
Parent variables				
Maternal age, years, mean (SD)	42.50 (4.27)	45.91 (6.14)	1.864	0.072
Maternal education, *n* (%)			0.330	0.566
Primary/Secondary school	6 (27.3)	2 (18.20)		
High school/University	16 (72.7)	9 (81.80)		
Paternal age, years, mean (SD)	47.68 (8.53)	48.55 (5.63)	0.303	0.764
Paternal education, *n* (%)			3.515	0.061
Primary/Secondary school	9 (40.90)	1 (9.10)		
High school/University	16 (59.10)	10 (90.90)		

PSG, protein supplemented group; CG, control group.

Parental variables regard the moment of the assessment.

Renal function was evaluated in 2 and 14 randomly selected CG and PSG children respectively (25% of each study group) at 6.3 years of age (SD 1.7) and resulted normal: mean creatinine value was 0.46 mg/dl (SD 0.06), mean urine pH was 5.9 (SD 0.8), mean urine specific molecular weight was 1018 (SD 10.5).

Assessment of anthropometric data showed no significant differences between PSG and CG weight mean Z-scores (Table 2). According to WHO definition, no children were classified as overweight nor obese.

**Table 2:** Mean z-score values calculated with world health organization curves in PSG and CG groups.

	PSG (*n* = 22)	CG (*n* = 11)	t	*p*
Weight	−0.062 (1.06)	0.038 (0.95)	0.265	0.793
Height	−0.483 (0.84)	−0.211 (0.84)	0.877	0.387
BMI	0.185 (1.19)	0.217 (1.12)	0.076	0.940

PSG, protein supplemented group; CG, control group.

Data are expressed as mean (SD) values. *p* value was generated by *t*-test.

The assessment of emotional and behavioral functioning showed specific profiles in children of the two groups. Specifically, PSG children reported a significantly higher score (worse) than CG ones when externalizing symptoms scales were measured by their mothers. Conversely, when externalizing scores were evaluated by fathers no significant differences in scores emerged between PSG and CG groups (Table 3).

**Table 3:** CBCL mean scores in mothers and fathers.

Respondents	PSG (*n* = 22)	CG (*n* = 11)	t	*p*
Mothers				
Internalizing symptoms	49.00 (11.05)	41.67 (9.43)	−1.954	0.059
Externalizing symptoms	48.48 (8.33)	41.83 (6.12)	−2.435	**0**.**020**
Fathers				
Internalizing symptoms	46.14 (9.45)	44.14 (11.39)	−0.540	0.593
Externalizing symptoms	40.45 (8.00)	40.83 (7.18)	−1.305	0.201

PSG, protein supplemented group; CG, control group.

Data are expressed as mean (SD) values. *p* value was generated by *t*-test. Significant differences are highlighted in bold.

Furthermore, a global homogeneity between groups was measured for internalizing scores, both when assessed by mothers and fathers (Table 3).

According to CBCL cut-off scores, no significant differences between PSG and CG emerged regarding the frequencies of clinical risk considering maternal perception of internalizing nor externalizing scores or in paternal perception of internalizing nor externalizing scores (all *p*s > 0.05) (Table 4).

**Table 4:** CBCL cut-off scores in mothers and fathers.

Respondents	PSG (*n* = 22)	CG (*n* = 11)	X2	*p*
Mothers				
Internalizing symptoms	3 (13.0)	0 (0.0)	1.712	0.191
Externalizing symptoms	1 (13.0)	0 (0.0)	0.537	0.464
Fathers				
Internalizing symptoms	2 (9.1)	2 (16.7)	0.429	0.512
Externalizing symptoms	0 (0.0)	0.0 (0.0)	//	//

PSG, protein supplemented group; CG, control group.

Data are expressed as *n* (%) values. *p* value was generated by X2.

## 4 Discussion

This preliminary study was conducted with the aim to evaluate long-term outcomes in a NICU population fed with human fortified milk.

Our main hypothesis was that protein enriched human milk fed to preterm newborns during the critical period of first weeks of life would have led to long term advantages in auxological and psychological outcome without concomitant metabolic consequences, such as obesity, during childhood (18, 19).

While our previous studies underlined the benefits on patients’ outcomes until 2 years of age (8, 10–12), in the present long-term evaluation the PSG children did not show any significant advantages compared to the standard protein intake group at neurodevelopmental level and psychological outcomes. Moreover, whereas some studies previously reported metabolic consequences, mainly obesity, in children born preterm and fed with high energy and protein intake, we did not find evidence of this in PSG group compared to CG sample.

The absence of long-term significant differences is consistent with a recent meta-analysis, which found no correlations between early nutritional supplementation—such as high protein intake—and increased incidence of later metabolic disease (20). The study of Lin et al. showed that supplemented groups had higher HDL concentration during childhood and a lower fasting blood glucose concentration than children feeding with lower protein intake: this difference seemed to vanish during adolescence. Childhood fasting blood glucose concentration is inversely related to pre-diabetes in adulthood. The meta-analysis substantially confirms that the early macronutrient supplementation in preterm and small for gestational age infants does not have adverse effect on metabolic outcome.

It is important to highlight that quick intrahospital growth rate may also be positively associated with the risk of future obesity and metabolic syndrome via epigenetic reprogramming (21). This is especially evident if the weight gain is accelerated; at age 8–11 the preterm children exhibit a greater risk of childhood obesity compared to those born at term. In contrast, the evidence linking rapid postnatal growth to later adiposity or cardiovascular disease risk factors in preterm children's results limited (22).

In the present study, children's BMI, corrected for gestational age at birth, was normal without significant statistical differences between the two CG and SPG groups. We did not find any causality between administration of an adequate protein content in human milk during the first periods of life and obesity in our preterm patients. According to parental report, all patients practiced at least one sport twice a week at the time of evaluation, excluding the possibility of cardiovascular fitness obfuscating interpretation of the results.

We further assessed the possible effects on neurobehavioral outcomes. From this perspective, aggressive nutritional intervention for “catch-up-growth” in preterm infants would appear largely justified. A systematic review, including observational studies, reported a positive association between postnatal weight or head growth and neurocognitive outcomes (22). Asuggesting that prenatal undernutrition turned out to be worse than postnatal rapid growth to determine neurodevelopmental outcomes.

Although Cochrane reports no benefits of fortification assessed beyond infancy based on limited available data (23), we have highlighted that the long-term benefits of extra-protein fortification were only present in fragile preterm infants (ELBW) aggressively fed during the short intrahospital period.

Premature infants weighing <1,000 g at birth may require more protein to grow compared to VLBW infants between 1,000 and 1,500 g. Enteral protein requirements likely need to be adjusted for growth as the infant develops, to accommodate for these differences, beginning with higher protein intake when the infant weighs <1,000 g with subsequent decreased amounts as weight and gestational age increase. Suitable growth, especially of lean body mass and particularly of the brain, is dependent on enrichment of human milk, well known for its beneficial effect, with adequate protein intake (24).

A final consideration regards the overall homogeneity in psychological outcomes. Our aim was driven by previous literature, which underlined that preterm children are at risk of emotional and behavioral problems, especially during the first years of life, that could also persist at school entry (25, 26). Regardless of nutrition, school-age could represent a sensitive time also for parents, induced by the expectations on children's performance, and by potential recollection of child's fragility. Consequent parental expectations could in turn represent a further pressure on children. We found no significant differences in internalizing or externalizing symptoms (in case of paternal evaluation). Differently from our hypothesis, this result suggests a global homogeneity between the groups and the lack of significant advantages reported during earlier infancy periods. Unexpectedly, the only significant differences were found when evaluations were performed by mothers and suggested that PSG children had more externalizing difficulties than CG children. This result would seem to suggest that not only the advantages of protein supplementation disappear, but they might even represent a risk factor. However, it should be underlined that these differences disappear when categorical scores are used, suggesting an absence of pathology in the sample, as reported in previous studies (26). So, the differences in maternal perception could be not considered as an indicator of relevant difficulties.

Nevertheless, the presence of differences regarding maternal perceptions could suggest that these mothers might experience some difficulties with their children, with potential negative consequences on the mother-child relationship. Although the inclusion of both parents in the assessment of their children should enable less biased evaluation of children, it is not clear whether differences are due to a more accurate assessment by mothers, given their usually higher involvement into children-care, or the presence of specific characteristic in women that influence their answers. The small sample size did not allow to include possible influence of specific characteristics of parent or of the relationship between parent and child.

A final relevant question concerns methodological issues: while we previously assessed infant outcomes by developmental scales (27), in the present research we chose to investigate a larger definition of psychological states, considering child emotional and behavioral functioning. These measures have the advantage to give a realistic perspective of the emotional state inside the families, but it could be possible that changes into these variables are weaker to detect compared to other outcomes, such as cognitive development. Moreover, it could be possible that cognitive outcomes could play a significant role in mediating the relationship between early intervention and parental perception. Indeed, according to the study by Lowe and colleagues (28), the level of cognitive, language and motor development could influence parental perception of their children. So, further studies, including both developmental scales and parental-report questionnaires, are suggested.

Future studies could help to further characterize these factors by, for example, adding the evaluation of the effect of fortified nutrition on cognitive development of preterm children once they reach school age.

The present results should be considered as preliminary, and several limitations of the study should be noted. First, the results need to be confirmed on larger samples. The limited sample size could have reduced the power of analyses and have prevented the testing of more sophisticated hypotheses and analyses. Second, in the present study all the variables are assessed by parental evaluation, while objective height and weight measures, as a psychological profile, are lacking. Further studies, including also structured and standardized assessment, are required. Last, future studies are needed to confirm the results also controlling for the effect of other variables. Indeed, specific nutritional characteristics (frequencies of breastfeeding/bottle maternal milk/bottle formula milk, months of breastfeeding, specific diets,…), as well as variables regarding children (i.e., gender, severity of prematurity as defined by the presence of Sepsis, IVH, NEC, PVL, ROP, …), parents (i.e., parental age, level of education,…) and environment could play a relevant role in influencing child outcomes and parental affective reactions and perceptions of the child.

Therefore, all these variables need to be considered for their possible influences on the outcomes to help to generalize results.

Despite preliminary, these findings might contribute to deepen the understanding of the long-term effects of neonatal enteral protein supplementation in VLBW preterm infants, suggesting that benefits are restricted to the neonatal term and first two years of age.

## Data Availability

The raw data supporting the conclusions of this article will be made available by the authors, without undue reservation.
